# Association between persistent musculoskeletal pain and incident sarcopenia in China: the mediating effect of depressive symptoms

**DOI:** 10.3389/fpubh.2024.1416796

**Published:** 2024-09-04

**Authors:** Shengliang Zhou, Yuan Liu, Yan Zhang, Naijia Luo, Quan Chen, Meiling Ge, Bin Shen

**Affiliations:** ^1^Department of Orthopedic Surgery and Orthopedic Research Institute, West China Hospital, Sichuan University, Chengdu, China; ^2^The Center of Gerontology and Geriatrics, National Clinical Research Center of Geriatrics, West China Hospital, Sichuan University, Chengdu, China

**Keywords:** sarcopenia, musculoskeletal pain, CHARLS, mediation analysis, depression

## Abstract

**Objective:**

To evaluate the association between musculoskeletal pain and incident sarcopenia and further explore the mediating effect of depressive symptoms among middle-aged and older Chinese adults.

**Methods:**

Using the data from the China Health and Retirement Longitudinal Study 2011 and 2015, we included 12,788 participants in the cross-sectional analysis and 8,322 for the longitudinal analysis. Musculoskeletal pains located in the neck, back, waist, shoulder, arm, wrist, leg, knee, and ankle were self-reported at baseline and follow-up. The diagnosis criteria of sarcopenia was based on the Asian Working Group for Sarcopenia 2019. Multivariable logistic regression models were used to evaluate the association between musculoskeletal pain, and the Karlson–Holm–Breen (KHB) method was used to explore the mediating effect of depressive symptoms.

**Results:**

Over the 4-year follow-up, 445 participants were identified with incident sarcopenia. In the longitudinal analysis, participants with baseline musculoskeletal pain (adjusted odds ratio (OR): 1.37, 95% confidence interval (CI): 1.07–1.76), persistent musculoskeletal pain (OR:1.68, 95%CI: 1.28–2.24), and persistent waist pain (OR:1.46, 95%CI: 1.04–2.03) were significantly associated with increased the risk of incident sarcopenia. Furthermore, depressive symptoms were found to partially mediate the association between musculoskeletal pain and incident sarcopenia.

**Conclusion:**

Persistent musculoskeletal pain, especially in waist area, was positively associated with a higher risk of sarcopenia among the middle-aged and older Chinese. Depressive symptoms played a partial mediating role in this association.

## Introduction

Sarcopenia, characterized as a generalized and progressive disease marked by the accelerated decline in muscle mass and function, has been identified as significant risk factor for adverse outcomes, such as falls, frailty, and even mortality (1). Sarcopenia has emerged as a severe public health concern. Although sarcopenia is strongly associated with the aging process, lifestyle factors and chronic disease also play critical role in the onset and progression of sarcopenia (2, 3). Epidemiological studies reveal that the global prevalence of sarcopenia varies from 10 to 27%, with evidence indicating a rapid escalation in the burden of sarcopenia, particularly within low-income and middle-income countries (4, 5). Therefore, it is essential to identify the modifiable risk factors that may lead to the development of sarcopenia.

Musculoskeletal pain is an unpleasant sensory and emotional experience typically indicative of tissue damage. Globally, musculoskeletal pain affects over 30% of the older adults (6). This condition has been associated with fragility, disability, and reduced mobility, all of which are known to play critical roles in the development and progression of sarcopenia (7, 8). However, recent studies have reported controversial findings regarding the association between musculoskeletal pain and sarcopenia (9–11). For instance, Lin et al. (9) and Veronese et al. (10) both found that pain significantly increased the risk of sarcopenia in the Chinese and English populations, respectively. Tsuji et al. found no association between low back and sarcopenia among the Japanese population (11). In addition, only the pain in the chest, low back, hip, or knee was considered in previous studies (9, 10). The other pain sites such as the arm, shoulder, and neck were ignored, which also accounted for a large proportion of the Chinese population (12). Therefore, the association between musculoskeletal pain and sarcopenia needs further exploration in the prospective studies.

Existing literature highlights the concurrent prevalence of musculoskeletal pain and depressive symptoms, with the former being a risk factor for the latter (13, 14). In addition, a notable prevalence of depression among patients with sarcopenia and a positive correlation between depressive symptoms and sarcopenia has been documented (15, 16). Thus, depression may be an important factor in the association between musculoskeletal pain and sarcopenia. However, no study has examined the potential mediating role of depression in the effects of musculoskeletal pain on the risk of incident sarcopenia.

Therefore, this study aimed to (1) further investigate the association between persistent musculoskeletal pain and sarcopenia and (2) explore the mediating role of depressive symptoms, using the data from the China health and retirement longitudinal study (CHARLS), which might provide clinical guidance for reducing sarcopenia risk among musculoskeletal pain adults.

## Methods

### Study population

The CHARLS is a comprehensive and ongoing longitudinal survey capturing a nationally representative sample of the Chinese population aged 45 years and above. CHARLS employs a multistage stratified probability-proportionate-to-size sampling method to select households from a wide geographic spread of 450 villages or residential communities across 28 provinces. This method ensures the demographic and geographic representativeness of the sample. The baseline survey, conducted in 2011, recruited a total of 17,708 participants across 10,257 households. All participants were interviewed using a standardized, structured questionnaire through face-to-face computer-assisted personal interviews (CAPI) by well-trained interviewers. Follow-up surveys were conducted every 2 years. The detailed description about the design of the CHARLS has been previously reported (17).

**Figure 1:**
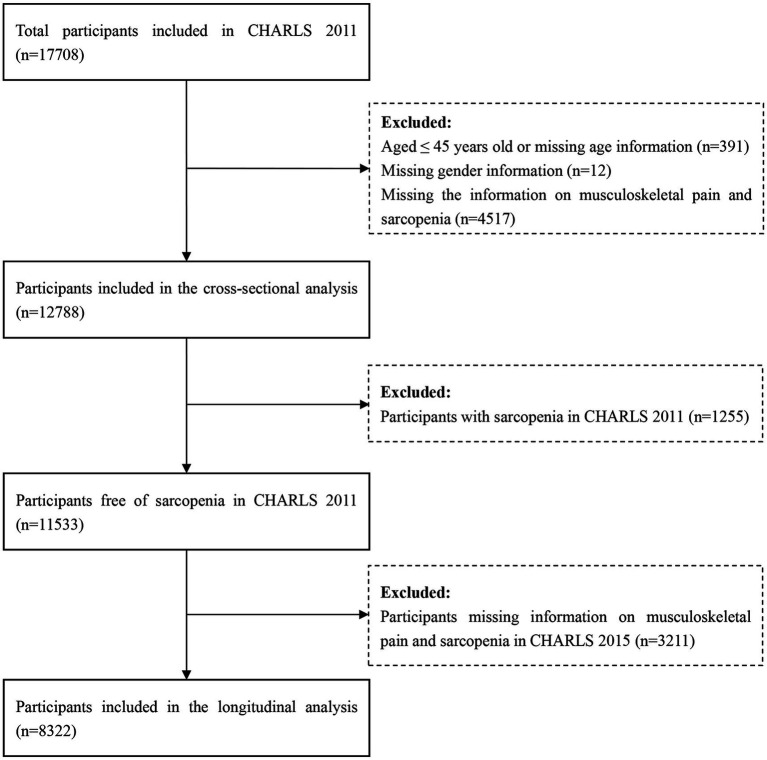
The flowchart of participants selection. CHARLS, China health and retirement longitudinal study.

In this study, we used the data from CHARLS 2011 and 2015. The analyses were divided into two sections: (1) In the cross-sectional analysis, the data of CHARLS 2011 was used. Finally, a total of 17,708 participants were interviewed. After excluding participants aged ≤45 years old or missing the age data (*N* = 391), missing the gender data (*N* = 12), missing the data on musculoskeletal pain and sarcopenia (*N* = 4,517), a total of 12,788 participants were included in the cross-sectional analysis. (2) In the longitudinal analysis, participants with sarcopenia in CHARLS 2011 (*N* = 1,255), and without the information about musculoskeletal pain and sarcopenia in CHARLS 2015 (*N* = 3,211) were further excluded. Our final analytical sample encompassed 8,322 participants, who were free from sarcopenia in CHARLS 2011 and had comprehensive follow-up in CHARLS 2015. The process of participants selection was illustrated in detail in Figure 1.

The written informed consents were obtained from all participants involved in the CHARLS, which received ethical approval from the Ethical Review Committee of Peking University (approval number: IRB00001052-11015). The CHARLS was conducted in alignment with the 1964 Declaration of Helsinki. Additionally, this study conformed to the Strengthening the Reporting of Observational Studies in Epidemiology (STROBE) reporting guidelines.

### Assessment of musculoskeletal pain

All participants were asked, “Are you often troubled with any body pains?” Those who responded “Yes” were then instructed to list all body parts where they were currently experiencing pain, including the head, neck, shoulder, arm, wrist, fingers, chest, stomach, back, waist, buttocks, leg, knees, ankle, and toes. In this study, any pain located in the neck, back, waist, shoulder, arm, wrist, leg, knee, or ankle was defined as musculoskeletal pain. Participants were identified as having persistent musculoskeletal pain if they reported experiencing musculoskeletal pain in both the CHARLS 2011 and CHARLS 2015 surveys.

### Assessment of sarcopenia

The assessment of sarcopenia in our study was based on the 2019 Asian Working Group for Sarcopenia (AWGS 2019) (18), focusing on three aspects: muscle strength, appendicular skeletal muscle mass (ASM), and physical performance. Sarcopenia was defined as low muscle mass plus low muscle strength and/or low physical performance. The evaluation of muscle strength was predominantly based on handgrip strength, using a standardized measurement protocol that has been previously reported (17). According to the AWGS 2019, low muscle strength is specifically identified by handgrip strength <28 kg for males and <18 kg for females. For the assessment of ASM, a validated anthropometric equation tailored to the Chinese population was applied, demonstrating significant alignment with dual energy X-ray absorptiometry (DXA) results in previous studies (19). The formula used was: ASM = 0.193 × weight (kg) + 0.107 × height (cm) – 4.157 × sex – 0.037 × age (years) – 2.631, where sex was coded as 1 for males and 2 for females. Measurements of body weight and height were conducted using precision instruments, and the skeletal muscle mass index (SMI) was calculated by dividing ASM by the square of height (ASM/height^2^). According to previous studies (20, 21), the threshold for low muscle mass was determined based on the lowest 20% of SMI within the study population, equating to <7.00 kg/m^2^ for males and <5.26 kg/m^2^ for females. Physical performance was assessed by the gait speed or the 5-time chair stand test, with thresholds for low physical performance set at a gait speed <1.0 m/s or a 5-time chair stand test ≥12 s.

### Assessment of depressive symptoms

The mediating variable in this study was depressive symptoms at baseline. Depressive symptoms were diagnosed using the 10-item Center for Epidemiologic Studies Depression Scale (CESD-10) at baseline. The CESD-10 has been proven to be a valid and reliable tool for screening depressive symptoms among Chinese community-dwelling older adult individuals (22). The CESD-10 questionnaire consists of 10 questions that evaluate feelings and behaviors experienced over the past week. Participants’ responses to each item were rated on a four-point scale, from “never or rarely” to “always,” with a composite score ranging from 0 to 30 (23, 24). A threshold score of 12 or above was indicative of depressive symptoms.

### Covariates

At baseline, the information of baseline demographic variables, health behaviors and chronic diseases of all participants were collected by the well-trained interviewers. The baseline demographic variables included age, gender, residence (urban, rural), marital status (married and living with spouse, others), and highest educational level (primary school or below, middle school, high school or above). Health behaviors included smoking status (current/ever/never smoking), drinking status (current/ever/never drinking), and body mass index (BMI). Chronic diseases included hypertension, diabetes mellitus (DM), depressive symptoms, dyslipidemia, cardiovascular disease, pulmonary disease, kidney disease, and arthritis. Hypertension was defined based on participants reporting a physician-diagnosed hypertension, or having a systolic blood pressure (BP) ≥ 140 mmHg or diastolic BP ≥ 90 mmHg. DM was defined based on participants reporting a physician-diagnosed diabetes, fasting glucose levels ≥7 mmol/L, random glucose levels ≥11.1 mmol/L, or glycosylated hemoglobin (HbA1c) ≥ 6.5%. Dyslipidemia was defined by either a ratio of cholesterol and high-density lipoprotein cholesterol (HDL-c) of ≥5.0 or self-reporting of a physician-diagnosed dyslipidemia. Cardiovascular disease, pulmonary disease, kidney disease, and arthritis were determined based on participants’ reported history of physician diagnoses.

### Statistical analysis

Continuous variables were presented as means ± SDs and categorical variables were presented as numbers with percentages. The differences in baseline characteristics between participants with and without musculoskeletal pain in the cross-sectional and longitudinal analyses were compared using Student’s *t*-test for continuous variables and chi-square test for categorical variables.

We used the multivariable logistic regression models to evaluated the association between baseline musculoskeletal pain and sarcopenia in the cross-sectional analysis, and mainly evaluated the association between persistent musculoskeletal pain and incident sarcopenia in the longitudinal analysis. The odds ratios (ORs) and 95% confidence intervals (CIs) were reported. Three models were used. Model 1 was a crude model. Model 2 adjusted for age, gender, residence, marital status, educational level, smoking status and drinking status. Model 3 further adjusted hypertension, DM, depressive symptoms, dyslipidemia, cardiovascular disease, pulmonary disease, kidney disease, and arthritis based on model 2. To exam the robustness of the findings, we conducted several sensitivity analyses. First, we conducted subgroup analyses stratified by age, gender, residence, hypertension, DM, depressive symptoms, and arthritis. Second, we additionally adjusted for the physical activity based on the model 3.

In addition, we employed the Karlson–Holm–Breen (KHB) method in Stata to evaluate the mediation effect of depressive symptoms on the association between musculoskeletal pain and incident sarcopenia. The KHB method was proposed by Kohler, Holm, and Breen (25), which could calculated the direct effect, indirect (mediating) effect, and the percentage of the mediating effect for the binary dependent or mediator variables.

All statistical analyses in this study were conducted using STATA/MP, version 17.0. The forest plot was created using EmpowerStats. The level of statistical significance was set at *p* < 0.05 (two-sided).

## Results

### Baseline characteristics in the cross-sectional and longitudinal analyses

The baseline characteristics of included participants based on musculoskeletal pain status in the cross-sectional analysis was presented in Table 1. The mean (SD) age was 59.39 (9.55) years old, and 6,690 (52.31%) were females. In the cross-sectional analysis, 3,857 (30.16%) participants had musculoskeletal pain. Participants with musculoskeletal pain seem to be older, female, living rural, married, having higher prevalence of hypertension, depressive symptoms, kidney disease, and arthritis compared to those without musculoskeletal pain (Table 1). Similar characteristics differences were observed in the longitudinal analysis between participants with and without persistent musculoskeletal pain (Supplementary Table S1).

**Table 1:** Baseline characteristics of included participants in the cross-sectional analysis by musculoskeletal pain.

	Total	Baseline musculoskeletal pain		*p*-value
		Yes	No	
Demographic characteristics	12,788	3,857 (30.16)	8,931 (69.84)	
Age, years	59.39 ± 9.55	59.87 ± 9.37	59.19 ± 9.62	<0.001
Gender, *n* (%)				<0.001
Male	6,098 (47.69)	1,468 (38.06)	4,630 (51.84)	
Female	6,690 (52.31)	2,389 (61.94)	4,301 (48.16)	
Residence, *n* (%)				<0.001
Urban	2,741 (21.49)	594 (15.43)	2,141 (24.11)	
Rural	10,014 (78.51)	3,256 (84.57)	6,758 (75.89)	
Marital status, *n* (%)				<0.001
Married and living with spouse	2,236 (17.49)	764 (19.81)	1,472 (16.48)	
Others	10,552 (82.51)	3,093 (80.19)	7,459 (83.52)	
Education level, *n* (%)				<0.001
Primary school or below	8,791 (68.74)	3,027 (78.48)	5,764 (64.54)	
Middle school	2,612 (20.43)	595 (15.43)	2017 (22.58)	
High school or above	1,385 (10.83)	235 (6.09)	1,150 (12.88)	
Health behavior				
Drinking, *n* (%)				<0.001
Current drinking	4,224 (33.05)	1,131 (29.33)	3,093 (34.66)	
Ever drinking	1,058 (8.28)	357 (9.26)	701 (7.85)	
Never drinking	7,499 (58.67)	2,368 (61.41)	5,131 (57.49)	
Smoking, *n* (%)				<0.001
Current drinking	3,936 (30.90)	1,047 (27.27)	2,889 (32.47)	
Ever drinking	1,121 (8.80)	318 (8.28)	803 (9.03)	
Never drinking	7,679 (60.29)	2,474 (64.44)	5,205 (58.50)	
BMI, kg/m^2^	23.47 ± 3.95	23.41 ± 4.12	23.49 ± 3.87	0.2649
Chronic diseases				
Hypertension, *n* (%)	5,218 (40.80)	1,627 (42.18)	3,591 (40.21)	0.037
Diabetes mellitus, *n* (%)	1,579 (12.38)	531 (13.80)	1,048 (11.76)	0.001
Depressive symptoms, *n* (%)	4,076 (31.87)	2018 (52.32)	2058 (23.04)	<0.001
CESD-10	8.35 ± 6.29	12.11 ± 6.58	6.77 ± 5.43	<0.001
Dyslipidemia, *n* (%)	2,737 (21.51)	898 (23.43)	1839 (20.69)	0.001
Cardiovascular disease, *n* (%)	1,478 (11.62)	632 (16.51)	846 (9.52)	<0.001
Pulmonary disease, *n* (%)	1,349 (10.59)	617 (16.11)	732 (8.22)	<0.001
Kidney disease, *n* (%)	812 (6.39)	448 (11.74)	364 (4.10)	<0.001
Arthritis, *n* (%)	4,364 (34.20)	2,181 (56.75)	2,183 (24.48)	<0.001
Handgrip strength, kg	32.64 ± 10.59	30.01 ± 9.84	33.78 ± 10.71	<0.001
ASM/height^2^, kg/m^2^	6.76 ± 1.56	6.56 ± 1.58	6.85 ± 1.54	<0.001
Gait speed, m/s	1.41 ± 4.63	1.38 ± 4.42	1.41 ± 4.73	0.787
5-time chair stand test, s	10.77 ± 4.38	11.65 ± 5.03	10.40 ± 4.01	<0.001
Sarcopenia, *n* (%)	1,255 (9.81)	460 (11.93)	795 (8.90)	<0.001

Continuous variables are presented as the mean ± standard deviation, and categorical variables are expressed as numbers (percentages). BMI, body mass index; CESD-10, 10-item Center for Epidemiologic Studies Depression Scale; ASM, appendicular skeletal muscle.

### Cross-sectional association between musculoskeletal pain and sarcopenia

In the cross-sectional analysis, the prevalence of sarcopenia in the total sample, musculoskeletal pain, no-musculoskeletal pain individuals was 9.81, 11.93, and 8.90%. The association between baseline musculoskeletal pain and sarcopenia was presented in Table 2. After adjusting for demographic, health behavior, and chronic diseases (Model 3), musculoskeletal pain were significantly associated with sarcopenia (OR: 1.17; 95%CI: 1.01–1.38). Additionally, we also observed that back pain, waist pain, shoulder pain, arm pain, wrist pain, knee pain, and ankle pain were all significantly associated with sarcopenia (Table 2).

**Table 2:** Odds ratios and 95% confidence intervals for sarcopenia by musculoskeletal pain in the cross-sectional analysis.

	Cases (%)	Model 1	*P*-value	Model 2	*P*-value	Model 3	*P*-value
Musculoskeletal pain							
Yes	460 (11.93)	1.38 (1.22–1.56)	<0.001	1.20 (1.04–1.37)	0.010	1.17 (1.01–1.38)	0.041
No	795 (8.90)	Ref		Ref		Ref	
Neck pain							
Yes	111 (12.20)	1.30 (1.05–1.60)	0.012	1.22 (0.97–1.55)	0.087	1.21 (0.93–1.56)	0.156
No	1,144 (9.63)	Ref		Ref		Ref	
Back pain							
Yes	172 (14.35)	1.62 (1.36–1.93)	<0.001	1.44 (1.18–1.75)	<0.001	1.42 (1.14–1.76)	0.001
No	1,083 (9.35)	Ref		Ref		Ref	
Waist pain							
Yes	308 (12.02)	1.33 (1.16–1.53)	<0.001	1.21 (1.03–1.41)	0.015	1.21 (1.01–1.43)	0.035
No	947 (9.26)	Ref		Ref		Ref	
Shoulder pain							
Yes	207 (12.64)	1.39 (1.18–1.63)	<0.001	1.29 (1.08–1.55)	0.004	1.35 (1.11–1.65)	0.003
No	1,048 (9.40)	Ref		Ref		Ref	
Arm pain							
Yes	192 (14.81)	1.71 (1.44–2.01)	<0.001	1.60 (1.32–1.93)	<0.001	1.68 (1.37–2.07)	<0.001
No	1,063 (9.25)	Ref		Ref		Ref	
Wrist pain							
Yes	102 (12.35)	1.32 (1.06–1.63)	0.012	1.26 (0.99–1.60)	0.058	1.30 (1.00–1.69)	0.047
No	1,153 (9.64)	Ref		Ref		Ref	
Leg pain							
Yes	250 (13.15)	1.48 (1.28–1.72)	<0.001	1.15 (0.97–1.36)	0.096	1.17 (0.97–1.41)	0.095
No	1,005 (9.23)	Ref		Ref		Ref	
Knee pain							
Yes	227 (13.00)	1.45 (1.24–1.69)	<0.001	1.18 (0.99–1.41)	0.051	1.23 (1.01–1.50)	0.034
No	9.31 (9.31)	Ref		Ref		Ref	
Ankle pain							
Yes	111 (14.76)	1.64 (1.33–2.03)	<0.001	1.41 (1.11–1.78)	0.005	1.48 (1.14–1.92)	0.003
No	1,144 (9.50)	Ref		Ref		Ref	

Data are presented as ORs (95% CIs). Model 1: crude model. Model 2: adjusted for age, gender, residence, marital status, educational level, drinking status, and smoking status. Model 3: further adjusted hypertension, DM, depressive symptoms, dyslipidemia, cardiovascular disease, pulmonary disease, kidney disease, and arthritis based on model 2. ORs, odds ratios; CIs, confidence intervals; DM, diabetes mellitus.

### Longitudinal association between persistent musculoskeletal pain and incident sarcopenia

**Figure 2:**
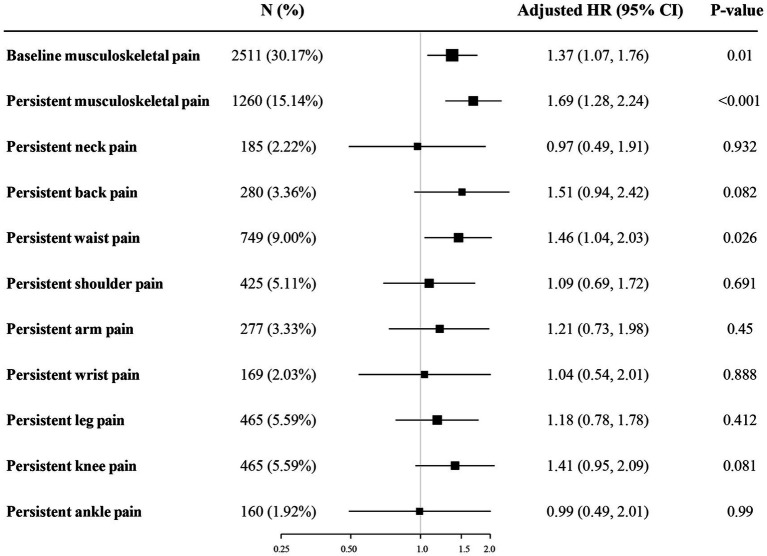
Odds ratios and 95% confidence intervals for incident sarcopenia by persistent musculoskeletal pain in the longitudinal analysis. The figure shows ORs and 95% CI for incident sarcopenia adjusted for age, gender, residence, marital status, educational level, drinking status, smoking status, hypertension, DM, depressive symptoms, dyslipidemia, cardiovascular disease, pulmonary disease, kidney disease, and arthritis. ORs, odds ratios; CIs, confidence intervals; DM, diabetes mellitus.

In the longitudinal analysis, a total of 8,322 participants without sarcopenia in the baseline were included, and 1,260 (15.14%) participants had persistent musculoskeletal pain. During the four-year follow-up period, 445 (5.35%) participants with incident sarcopenia were identified, of which 104 (8.25%) participants had persistent musculoskeletal pain and 341 (4.83%) participants did not have. The association between musculoskeletal pain and incident sarcopenia was presented in Table 3 and Figure 2. After adjusting for covariates in model 3, baseline musculoskeletal pain (OR:1.37; 95%CI: 1.07–1.76), persistent musculoskeletal pain (OR:1.69; 95%CI: 1.28–2.24), and persistent waist pain (OR:1.46; 95%CI: 1.04–2.03) were significantly associated with increased risk of incident sarcopenia. Other sites of persistent musculoskeletal pain were not observed significant association with sarcopenia. The subgroup analyses were presented in Supplementary Table S2. The longitudinal association between persistent musculoskeletal pain and incident sarcopenia did not significantly change after further adjusting for physical activity in Supplementary Table S3.

**Table 3:** Odds ratios and 95% confidence intervals for incident sarcopenia by persistent musculoskeletal pain in the longitudinal analysis.

	Cases (%)	Model 1	*P*	Model 2	*P*	Model 3	*P*
Baseline musculoskeletal pain							
Yes	175 (6.97)	1.53 (1.26–1.87)	<0.001	1.42 (1.15–1.75)	0.001	1.37 (1.07–1.76)	0.010
No	270 (4.65)	Ref		Ref		Ref	
Persistent musculoskeletal pain							
Yes	104 (8.25)	1.77 (1.41–2.22)	<0.001	1.71 (1.33–2.19)	<0.001	1.69 (1.28–2.24)	<0.001
No	341 (4.83)	Ref		Ref		Ref	
Persistent neck pain							
Yes	12 (6.49)	1.23 (0.68–2.23)	0.487	1.16 (0.62–2.21)	0.628	0.97 (0.49–1.91)	0.932
No	433 (5.32)	Ref		Ref		Ref	
Persistent back pain							
Yes	27 (9.64)	1.94 (1.29–2.92)	0.001	1.75 (1.13–2.71)	0.011	1.51 (0.94–2.42)	0.082
No	418 (5.20)	Ref		Ref		Ref	
Persistent waist pain							
Yes	59 (7.88)	1.59 (1.19–2.11)	0.001	1.53 (1.13–2.08)	0.006	1.46 (1.04–2.03)	0.026
No	386 (5.10)	Ref		Ref		Ref	
Persistent shoulder pain							
Yes	31 (7.29)	1.42 (0.97–2.07)	0.068	1.39 (0.92–2.08)	0.109	1.09 (0.69–1.72)	0.691
No	414 (5.24)	Ref		Ref		Ref	
Persistent arm pain							
Yes	23 (8.30)	1.63 (1.05–2.53)	0.028	1.28 (0.80–2.05)	0.294	1.21 (0.73–1.98)	0.450
No	422 (5.25)	Ref		Ref		Ref	
Persistent wrist pain							
Yes	13 (7.69)	1.48 (0.83–2.64)	0.174	1.26 (0.68–2.32)	0.449	1.04 (0.54–2.01)	0.888
No	432 (5.30)	Ref		Ref		Ref	
Persistent leg pain							
Yes	36 (7.74)	1.52 (1.07–2.17)	0.019	1.22 (0.83–1.78)	0.303	1.18 (0.78–1.78)	0.412
No	409 (5.21)	Ref		Ref		Ref	
Persistent knee pain							
Yes	42 (9.03)	1.83 (1.31–2.56)	<0.001	1.51 (1.05–2.16)	0.023	1.41 (0.95–2.09)	0.081
No	403 (5.13)	Ref		Ref		Ref	
Persistent ankle pain							
Yes	11 (6.88)	1.31 (0.71–2.44)	0.387	0.98 (0.51–1.91)	0.97	0.99 (0.49–2.01)	0.990
No	434 (5.32)	Ref		Ref		Ref	

Data are presented as ORs (95% CIs). Model 1: crude model. Model 2: adjusted for age, gender, residence, marital status, educational level, drinking status, and smoking status. Model 3: further adjusted hypertension, DM, depressive symptoms, dyslipidemia, cardiovascular disease, pulmonary disease, kidney disease, and arthritis based on model 2. ORs, odds ratios; CIs, confidence intervals; DM, diabetes mellitus.

### Mediation analysis

In the longitudinal analysis, participants who developed sarcopenia exhibited significantly higher CESD-10 scores compared to those who did not develop sarcopenia, with mean scores of 10.41 ± 6.68 and 8.08 ± 6.17, respectively. Table 4 showed the potential mediating effects of depressive symptoms between musculoskeletal pain and incident sarcopenia, employing the KHB method. After adjusting for covariates, depressive symptoms were found to mediate 22.65, 15.43, and 22.42% of the total effects in the associations between baseline musculoskeletal pain, persistent musculoskeletal pain, and persistent waist pain with the incident sarcopenia, respectively.

**Table 4:** Mediation effects of depressive symptoms using KHB mediation analyses.

Mediation of depressive symptoms	Effects, OR (95% CI)			Confounding percentage
	Total effect	Direct effect	Indirect effect	
Baseline musculoskeletal pain	1.44 (1.15–1.81)^**^	1.33 (1.05–1.68)^*^	1.08 (1.02–1.15)^*^	22.65%
Persistent musculoskeletal pain	1.77 (1.36–2.31)^***^	1.62 (1.23–2.13)^***^	1.09 (1.01–1.17)^*^	15.43%
Persistent waist pain	1.57 (1.13–2.16)^**^	1.41 (1.02–1.97)^*^	1.10 (1.03–1.18)^**^	22.42%

The mediation analyses all adjusted for age, gender, residence, marital status, educational level, drinking status, smoking status, hypertension, DM, depressive symptoms, dyslipidemia, cardiovascular disease, pulmonary disease, kidney disease, and arthritis. ORs, odds ratios; CIs, confidence intervals; DM, diabetes mellitus. *p-value < 0.5, **p-value < 0.01, ***p-value < 0.001.

## Discussion

By using the data from a nationally representative sample of middle-aged and older Chinese adults, we found the presence of musculoskeletal pain was associated with higher prevalence of sarcopenia in the cross-sectional study. We also found the persistent musculoskeletal pain, especially when located in the waist, was significantly associated with an increased risk of incident sarcopenia. In addition, our findings indicated that depressive symptoms partially mediated this association between musculoskeletal and incident sarcopenia.

Persistent musculoskeletal pain is a prevalent condition among older adults, leading to substantial disability and imposing significant costs on both individuals and society (26). Our findings revealed a positive association between persistent musculoskeletal pain and incident sarcopenia, which were in line with previous studies. Veronese et al. (10) observed that pain was associated with higher risk of incident sarcopenia over 10-year follow-up period among the English population. Similarly, Lin et al. (9) found analogous results in a Chinese population following a 1-year follow-up study. Our research contributes novel insights to the existing body of literature by examining the impact of persistent pain on the development of sarcopenia. Moreover, unlike prior studies that primarily focused on pain in the knee, hip, low back, and feet, our study broadened the scope to include a wider range of musculoskeletal pain sites and investigated their association with sarcopenia. We found that persistent waist pain was significantly associated with an increased risk of incident sarcopenia, a connection not observed with pain in other body areas. These findings were inconsistent with previous studies. In prior studies, low back pain was associated with the incident sarcopenia (9, 10). This discrepancy was attributed that the CHALRS did not distinguish low back pain as a separate category and waist pain could encompass a range of symptoms that might be classified under low back pain in others studies. In addition, a study reported that knee pain also could elevate the risk of sarcopenia (10). However, this study was conducted in the English older population and did not address the effects of persistent knee pain.

The association between musculoskeletal pain and sarcopenia may be elucidated through several underlying mechanisms. First, existing literature indicated that chronic musculoskeletal pain is associated with an elevated risk of disability and sedentary behavior, both of which significantly reduce physical activity levels (27, 28). Reduced physical activity is a recognized risk factor for the development of sarcopenia (3, 29). Second, persistent musculoskeletal pain may trigger a series of inflammatory responses, elevating the levels of pro-inflammatory factors, including interleukin-1 beta (IL-1β) and tumor necrosis factor alpha (TNFα) (30). Elevated levels of these pro-inflammatory factors have the potential to accelerate muscle loss by enhancing protein degradation and decreasing protein synthesis, thus contributing to the onset and progression of sarcopenia (31). Additionally, pharmacological interventions are commonly employed in the management of chronic pain, with non-steroidal anti-inflammatory drugs (NSAIDs) and opioids frequently prescribed. The administration of these medications has been strongly correlated with muscle loss. The use of NSAIDs has been linked to adverse effects on muscle tissue, potentially exacerbating muscle degradation (32). Opioids, on the other hand, act on the central nervous system to alleviate pain but can lead to decreased physical activity and subsequent muscle atrophy (33).

Notably, pain in the waist region, compared to other musculoskeletal areas, significantly impairs core movement and stability. The waist functions as a critical fulcrum for body mechanics, bearing the upper body’s weight and facilitating a broad range of movements indispensable for daily functioning. Persistent waist pain can severely limit not only specific exercises aimed at maintaining muscle mass but also routine physical activities that are crucial for preserving muscle function and preventing atrophy (34). Furthermore, the Global Burden of Disease 2010 study data highlighted that waist pain was the leading cause of disability globally, surpassing all other conditions (35). Additionally, several studies have indicated that waist pain is frequently associated with muscle atrophy and may be a risk factor for muscle atrophy (36, 37). The correlation between chronic waist pain and muscle degradation suggests that effective management of waist pain is critical not only for pain relief but also for the prevention of sarcopenia and the maintenance of overall musculoskeletal health.

Based on the findings of mediation analyses, we found that depressive symptoms partially mediated the association between baseline musculoskeletal pain, persistent musculoskeletal pain, and persistent waist pain with incident sarcopenia, attenuating the association by 22.65, 15.43, and 22.42%, respectively. To our knowledge, no prior study had investigated the mediating role of depressive symptoms on the relationship between musculoskeletal pain and sarcopenia. It is well-documented that individuals suffering chronic musculoskeletal pain frequently experience psychological distress, potentially resulting in depression (38). Moreover, depression itself is recognized as a risk factor for the musculoskeletal pain (39). Individuals with depression often experience the changed behavior and dietary habits. Depression is significantly associated with reduced physical activity and decreased dietary intake, which contributed to the incident of sarcopenia (15, 16). Furthermore, both sarcopenia and depression have been linked to the presence of low-grade inflammation and oxidative stress, suggesting a shared pathophysiological pathway (40). Depression also contributes to decreased social participation and leads to social isolation, which has been associated with an increased risk of incident sarcopenia (41). Our findings suggest a multidirectional relationship between musculoskeletal pain, depressive symptoms, and sarcopenia, underscoring the importance of considering psychological well-being in the management of musculoskeletal pain and prevention of sarcopenia.

This study has several strengths. First, this study was conducted employing a large and nationally representative sample, enabling the findings to be applicable to the middle-age and older population in China. Second, this study offers new evidence on how musculoskeletal pain in various locations contributes to sarcopenia risk. In addition, we firstly explored the role of depressive symptoms as a mediator in the association between musculoskeletal pain and incident sarcopenia, emphasizing the importance of the mental health in the routine care for patients suffering chronic pain. However, there are several limitations should be noted. First, consistent with previous studies (21, 42, 43), the assessment of muscle mass was estimated by a measured formula rather than using the DXA or bioelectrical impedance analysis. Consequently, although the formula has been validated in Chinese population and showed good consistency with DXA, the diagnoses of sarcopenia might be subject to a degree of bias (19). Second, the CHARLS did not collect the information on the medications for musculoskeletal pain, such as NSAIDs, opioids and antidepressants. Antidepressants are often recommended for the management of musculoskeletal pain (44). Although recent evidence indicated that the effects of antidepressants on musculoskeletal pain are small, the lack of data on antidepressant use may potentially mediate the association between musculoskeletal pain, depressive symptoms, and sarcopenia (45). Therefore, future studies should further include comprehensive data on the use of the medications to better understand their impacts on this complex interplay. Third, the assessment of musculoskeletal pain was based on self-reported data, which may affect the accuracy of the findings. In addition, although the study has adjusted for multiple confounding variables, it did not encompass all potential confounders, such as nutritional status and dietary intake.

## Conclusion

In conclusion, our findings indicate that the persistent musculoskeletal pain, especially in waist area, was significantly associated with incident sarcopenia among the middle-aged and older Chinese. Moreover, this association was partially mediated by depressive symptoms. These results highlight the essential role of mental health considerations in the management of musculoskeletal pain and in the prevention of sarcopenia.

## Data Availability

The datasets presented in this study can be found in online repositories. The names of the repository/repositories and accession number(s) can be found in the article/Supplementary material.
