# Supplementary material for: Association between persistent musculoskeletal pain and incident sarcopenia in China: the mediating effect of depressive symptoms
Source: Front Public Health. 2024 Sep 4;12:1416796. doi: 10.3389/fpubh.2024.1416796 (PMC11408356; doi:10.3389/fpubh.2024.1416796)
Supplement: Supplementary file 1 [file Table_1.DOCX]

**Table S1** Baseline characteristics of included participants in the longitudinal analysis by persistent musculoskeletal pain.

|  | **Total** | **Persistent musculoskeletal pain** | | **P-value** |
| --- | --- | --- | --- | --- |
|  |  | **Yes** | **No** |  |
| N | 8322 | 1260 (15.14) | 7062 (84.86) |  |
| **Demographic** |  |  |  |  |
| Age, years | 58.02 ± 8.39 | 58.42 ± 7.99 | 57.95 ± 8.45 | 0.065 |
| Gender |  |  |  | <0.001 |
| Male | 3949 (47.45) | 402 (31.90) | 3547 (50.23) |  |
| Female | 4373 (52.55) | 858 (68.10) | 3515 (49.77) |  |
| Residence |  |  |  | <0.001 |
| Urban | 1471 (17.72) | 152 (12.06) | 1319 (18.73) |  |
| Rural | 6831 (82.28) | 1108 (87.94) | 5723 (81.27) |  |
| Marital status |  |  |  | 0.001 |
| Married and living with spouse | 1260 (15.14) | 1041 (14.59) | 219 (18.42) |  |
| Others | 7062 (84.86) | 6092 (85.41) | 970 (81.58) |  |
| Education level |  |  |  | <0.001 |
| Primary school or below | 5708 (68.59) | 1021 (81.03) | 4687 (66.37) |  |
| Middle school | 1776 (21.34) | 167 (13.25) | 1609 (22.78) |  |
| High school or above | 838 (10.07) | 72 (5.71) | 766 (10.85) |  |
| **Health behavior** |  |  |  |  |
| Drinking |  |  |  | <0.001 |
| Current drinking | 2788 (33.52) | 331 (26.27) | 2457 (34.81) |  |
| Ever drinking | 644 (7.74) | 106 (8.41) | 538 (7.62) |  |
| Never drinking | 4886 (58.74) | 823 (65.32) | 4063 (57.57) |  |
| Smoking |  |  |  | <0.001 |
| Current drinking | 2564 (30.87) | 292 (23.23) | 2272 (32.23) |  |
| Ever drinking | 700 (8.43) | 91 (7.24) | 609 (8.64) |  |
| Never drinking | 5043 (60.71) | 874 (69.53) | 4169 (59.3) |  |
| BMI, kg/m^2^ | 23.93 ± 3.81 | 23.98 ± 3.97 | 23.93 ± 3.78 | 0.618 |
| **Chronic diseases** |  |  |  |  |
| Hypertension | 3262 (39.20) | 530 (42.06) | 2732 (38.69) | 0.024 |
| Diabetes mellitus | 1025 (12.34) | 178 (14.14) | 847 (12.01) | 0.035 |
| Depressive symptoms | 2627 (33.09) | 755 (63.93) | 1872 (27.70) | <0.001 |
| Dyslipidemia | 1924 (23.13) | 328 (26.03) | 1596 (22.61) | 0.008 |
| Cardiovascular disease | 905 (10.94) | 235 (18.85) | 670 (9.54) | <0.001 |
| Pulmonary disease | 815 (9.83) | 219 (17.46) | 596 (8.47) | <0.001 |
| Kidney disease | 542 (6.56) | 192 (15.37) | 350 (4.99) | <0.001 |
| Arthritis | 2857 (34.40) | 774 (61.58) | 2083 (29.55) | <0.001 |
| Incident sarcopenia | 445 (5.35) | 104 (8.25) | 341 (4.83) | <0.001 |

Continuous variables are presented as the mean ± standard deviation, and categorical variables are expressed as numbers (percentages).

Abbreviations: BMI, body mass index; ASM, appendicular skeletal muscle.

**Table S2** Odds ratios and 95% confidence intervals for incident sarcopenia by persistent musculoskeletal pain in subgroup analyses.

| **Subgroups** | | **Baseline musculoskeletal pain** | **Persistent musculoskeletal pain** | **Persistent back pain** | **Persistent waist pain** | **Persistent shoulder pain** | **Persistent arm pain** | **Persistent leg pain** | **Persistent knee pain** |
| --- | --- | --- | --- | --- | --- | --- | --- | --- | --- |
| **Age** | |  |  |  |  |  |  |  |  |
| ≤60 | | 1.17 (0.73-1.86) | 1.25 (0.73-2.14) | 0.64 (0.19-2.13) | 1.28 (0.69-2.38) | 0.50 (0.17-1.43) | 1.08 (0.41-2.83) | 0.95 (0.41-2.20) | 1.81 (0.90-3.61) |
| >60 | | 1.38 (1.04-1.82) | 1.64 (1.19-2.25) | 1.69 (1.01-2.84) | 1.35 (0.92-1.99) | 1.24 (0.75-2.05) | 1.34 (0.76-2.36) | 1.33 (0.84-2.09) | 1.21 (0.77-1.91) |
| **Gender** | |  |  |  |  |  |  |  |  |
| Male | | 1.32 (0.92-1.88) | 1.92 (1.24-2.97) | 2.01 (0.94-4.27) | 1.63 (0.95-2.80) | 1.75 (0.84-3.62) | 0.91 (0.31-2.71) | 1.59 (0.78-3.24) | 1.48 (0.76-2.91) |
| Female | | 1.44 (1.02-2.04) | 1.60 (1.11-2.31) | 1.37 (0.75-2.52) | 1.37 (0.89-2.10) | 0.85 (0.47-1.54) | 1.32 (0.75-2.32) | 1.05 (0.63-1.74) | 1.38 (0.84-2.25) |
| **Residence** | |  |  |  |  |  |  |  |  |
| Urban | | 1.64 (0.66-4.06) | 1.54 (0.47-4.95) | 4.34 (0.69-26.98) | 1.63 (0.40-6.66) | 1.54 (0.29-8.16) | 1.18 (0.12-11.12) | 3.01 (0.65-13.81) | 2.51 (0.57-10.93) |
| Rural | | 1.36 (1.05-1.75) | 1.72 (1.29-2.29) | 1.41 (0.87-2.30) | 1.47 (1.04-2.07) | 1.07 (0.66-1.71) | 1.23 (0.74-2.04) | 1.11 (0.72-1.69) | 1.39 (0.92-2.08) |
| **Hypertension** | |  |  |  |  |  |  |  |  |
| Yes | | 1.48 (0.99-2.21) | 1.66 (1.05-2.63) | 1.61 (0.76-3.37) | 1.42 (0.82-2.46) | 1.28 (0.63-2.58) | 1.24 (0.56-2.70) | 1.15 (0.61-2.13) | 0.78 (0.38-1.59) |
| No | | 1.37 (1.01-1.88) | 1.76 (1.24-2.52) | 1.41 (0.75-2.62) | 1.49 (0.97-2.28) | 0.94 (0.51-1.72) | 1.12 (0.58-2.15) | 1.25 (0.72-2.17) | 1.97 (1.22-3.17) |
| **Diabetes** | |  |  |  |  |  |  |  |  |
| Yes | | 2.05 (1.02-4.12) | 2.08 (0.96-4.48) | 1.94 (0.56-6.74) | 1.91 (0.80-4.53) | 1.67 (0.50-5.59) | 0.69 (0.14-3.34) | 1.40 (0.46-4.29) | 1.78 (0.66-4.76) |
| No | | 1.31 (1.01-1.71) | 1.66 (1.23-2.25) | 1.52 (0.91-2.54) | 1.39 (0.96-2.00) | 1.04 (0.64-1.71) | 1.34 (0.79-2.26) | 1.20 (0.77-1.87) | 1.36 (0.88-2.10) |
| **Depressive symptoms** |  | |  |  |  |  |  |  |  |
| Yes | | 1.46 (1.02-2.09) | 1.39 (0.97-2.00) | 1.70 (1.01-2.88) | 1.27 (0.84-1.93) | 1.22 (0.72-2.05) | 1.18 (0.65-2.15) | 0.88 (0.54-1.45) | 1.17 (0.72-1.90) |
| No | | 1.28 (0.90-1.82) | 2.28 (1.48-3.51) | 0.86 (0.24-2.92) | 1.88 (1.07-3.30) | 0.78 (0.27-2.21) | 1.42 (0.57-3.51) | 2.36 (1.17-4.75) | 2.15 (1.09-4.20) |
| **Arthritis** | |  |  |  |  |  |  |  |  |
| Yes | | 1.18 (0.77-1.62) | 1.42 (0.97-2.08) | 1.40 (0.78-2.51) | 1.32 (0.85-2.04) | 1.04 (0.60-1.80) | 0.93 (0.51-1.69) | 1.14 (0.70-1.88) | 1.24 (0.80-2.04) |
| No | | 1.61 (1.17-2.23) | 2.10 (1.40-3.15) | 1.81 (0.81-4.08) | 1.79 (1.01-2.89) | 1.03 (0.44-2.39) | 2.17 (0.91-5.19) | 1.26 (0.58-2.58) | 1.73 (0.82-3.62) |

Data are presented as ORs (95 % CIs).

The subgroups analyses were adjusted for age, gender, residence, marital status, educational level, drinking status, smoking status, hypertension, DM, depressive symptoms, dyslipidemia, cardiovascular disease, pulmonary disease, kidney disease, and arthritis.

Abbreviations: ORs, odds ratios; CIs, confidence intervals; DM, diabetes mellitus.

**Table S3** Odds ratios and 95% confidence intervals for incident sarcopenia by musculoskeletal pain after further adjusting for physical activity (N=3614)

|  | **Model 4** | **P** | **Model 5** | **P** |
| --- | --- | --- | --- | --- |
| Baseline musculoskeletal pain | 1.36 (1.08-1.71) | 0.001 | 1.33 (1.02-1.74) | 0.031 |
| Persistent musculoskeletal pain | 1.60 (1.23-2.09) | 0.001 | 1.67 (1.23-2.26) | 0.001 |
| Persistent waist pain | 1.44 (1.03-2.01) | 0.029 | 1.50 (1.04-2.17) | 0.029 |

Data are presented as ORs (95 % CIs).

Model 4: adjusted for age, gender, residence, marital status, educational level, drinking status, smoking status, and physical activity.

Model 5: further adjusted for hypertension, DM, depressive symptoms, dyslipidemia, cardiovascular disease, pulmonary disease, kidney disease, and arthritis based on Model 4.

The degree of physical activity was assessed by the International Physical Activity Questionnaire.

Abbreviations: ORs, odds ratios; CIs, confidence intervals; DM, diabetes mellitus.
